# The Impact of Sex, Circadian Disruption, and the Clock^Δ19/Δ19^ Genotype on Alcohol Drinking in Mice

**DOI:** 10.3390/genes13040701

**Published:** 2022-04-15

**Authors:** Abanoub Aziz Rizk, Bryan W. Jenkins, Yasmine Al-Sabagh, Shahnaza Hamidullah, Cristine J. Reitz, Mina Rasouli, Tami A. Martino, Jibran Y. Khokhar

**Affiliations:** Department of Biomedical Sciences, Ontario Veterinary College, University of Guelph, Guelph, ON N1G 2W1, Canada; aazizriz@uoguelph.ca (A.A.R.); bjenki01@uoguelph.ca (B.W.J.); yalsabag@uoguelph.ca (Y.A.-S.); shamidul@uoguelph.ca (S.H.); cristine.reitz@utoronto.ca (C.J.R.); mrasouli@uoguelph.ca (M.R.); tmartino@uoguelph.ca (T.A.M.)

**Keywords:** shiftwork, sex differences, alcohol use disorder, chronobiology, chronicity

## Abstract

Shift work is associated with increased alcohol drinking, more so in males than females, and is thought to be a coping mechanism for disrupted sleep cycles. However, little is presently known about the causal influence of circadian rhythm disruptions on sex differences in alcohol consumption. In this study, we disrupted circadian rhythms in female and male mice using both environmental (i.e., shifting diurnal cycles) and genetic (i.e., Clock^Δ19/Δ19^ mutation) manipulations, and measured changes in alcohol consumption and preference using a two-bottle choice paradigm. Alcohol consumption and preference, as well as food and water consumption, total caloric intake, and weight were assessed in adult female and male Clock^Δ19/Δ19^ mutant mice or wild-type (WT) litter-mates, housed under a 12-hour:12-hour light:dark (L:D) cycle or a shortened 10-hour:10-hour L:D cycle. Female WT mice (under both light cycles) increased their alcohol consumption and preference over time, a pattern not observed in male WT mice. Compared to WT mice, Clock^Δ19/Δ19^ mice displayed increased alcohol consumption and preference. Sex differences were not apparent in Clock^Δ19/Δ19^ mice, with or without shifting diurnal cycles. In conclusion, sex differences in alcohol consumption patterns are evident and increase with prolonged access to alcohol. Disrupting circadian rhythms by mutating the *Clock* gene greatly increases alcohol consumption and abolishes sex differences present in WT animals.

## 1. Introduction

Increases in problematic alcohol use are associated with shift work. Thought to develop as a coping mechanism for the loss of quality sleep that shift workers experience [1], the odds of short-term alcohol consumption are increased in shift workers while the odds of daily drinking are reduced, possibly reflecting a pattern of increased binge drinking behaviour; female shift workers reportedly consumed less alcohol than male shift workers, although studied cohorts were biased toward male subjects [2,3]. Increased alcohol consumption is reported in Tunisian and Japanese shift workers compared to workers on standard schedules, and these reports further implicate poor sleep as a contributing factor [4,5]. Contrary evidence also exists, however, from a population of Norwegian nurses demonstrating that shift work is associated with lower self-reported scores on the Alcohol Use Disorders Identification Test—Consumption [6] and from a population of Finnish nurses demonstrating that shift work is not associated with altered alcohol consumption [7]. Although alcohol consumption may be a sleep-loss coping mechanism that leads to problematic, binge drinking behaviour, divergent circadian biology may also contribute to alcohol consumption and alcohol use disorder [8]. To reconcile the contradictory findings and understand the biological mechanisms linking shift work and alcohol consumption, rodent models are often used to investigate the individual and combined effects of environmental and biological circadian rhythm disruptions.

Produced in the suprachiasmatic nucleus (SCN) of the anterior hypothalamus, the circadian rhythm programs neuronal, hormonal, and physiological processes for optimal temporal efficiency, and receives input from sensory systems to update internal processes against external diurnal cycling [9]. This internal rhythm entrains to a light:dark (L:D) period of 24-hours (12:12 L:D). Disruptions in diurnal cycling desynchronize our internal circadian rhythm from the external environment, altering circadian gene expression and producing negative health consequences such as disrupted sleep [10,11,12]. Comparing male C57Bl/6 mice entrained to either a shortened (6:18) or lengthened (18:6) L:D cycle with continuous access to 10% ethanol via a two-bottle choice paradigm reveals that mice on the shortened diurnal cycle have increased alcohol consumption and preference, compared to those on the lengthened diurnal cycle [13]. However, in female and male high- and low-alcohol preferring mice entrained to a shortened (11:11), lengthened (13:13), or standard (12:12) L:D cycle, with continuous access to 10% ethanol via the two-bottle choice paradigm, mice on the shifted diurnal cycles showed decreased alcohol consumption compared to 12:12 L:D mice; however, this failed to achieve statistical significance [14]. Decreased alcohol consumption is also apparent in female and male inbred Fischer and Lewis rats with continuous access to 10% ethanol via the two-bottle choice paradigm and entrained to a standard (12:12) or shortened (6:6) L:D cycle. All 6:6 L:D rats displayed statistically significant reductions in alcohol consumption, while only the 6:6 L:D Fischer rats had reduced alcohol preferences [15]. Although this evidence demonstrates that shifting diurnal cycles produces measurable effects on alcohol consumption and preference, genetically modifying mice to disrupt biological mechanisms involved in circadian rhythmicity also produces a reliable model for investigating the association between circadian disruptions and alcohol consumption.

The transcriptional activator gene Circadian Locomotor Output Cycles Kaput (CLOCK or Clock) is essential for circadian rhythms, as it is responsible for generating down-stream transcription factors that self-regulate to produce a biochemical rhythmicity [16]. Thus, mutating the *Clock* gene in mice permits the study of causal contributions to circadian rhythms and their synchronization with external factors [17], and is commonly performed via the Clock^Δ19/Δ19^ point mutation, an adenine-to-thymine transversion that results in the deletion of 51 amino acids from the Clock protein [18]. Mice with the Clock^Δ19/Δ19^ mutation sleep less than wild-type (WT) mice and have extended circadian periodicity that becomes arrhythmic over time, which is thought to be reflective of circadian misalignment resulting from shift work schedules [19,20,21,22]. While various studies show that alcohol exposure disrupts circadian biology in mice [23,24,25,26], to date only one study has directly investigated alcohol consumption in Clock^Δ19/Δ19^ mice. Providing female and male Clock^Δ19/Δ19^ mice and WT controls with continuous access to 10% ethanol via the two-bottle choice paradigm reveals that Clock^Δ19/Δ19^ mice consume more alcohol than WT controls, with female Clock^Δ19/Δ19^ mice demonstrating a statistically significant effect. Knocking down *Clock* expression in WT mice using RNA interference increased alcohol consumption to levels observed in Clock^Δ19/Δ19^ mice [27]. Unfortunately, this study did not compare the sexes, even though male Clock^Δ19/Δ19^ mice did not demonstrate a statistically significant difference in alcohol consumption from WT controls.

Although not fully understood, sex differences in circadian biology are apparent in the divergent morphology of the rodent SCN, and in differential responses to sleep/wake or rest/active cycle disturbances [28], including shifting diurnal cycles. Female and male high alcohol-drinking rats with continuous access to 10% ethanol via the two-bottle choice paradigm were entrained to either a standard (12:12) L:D cycle or shortened 6-hour (6:6) L:D cycle. Female 12:12 L:D rats decreased, whereas female 6:6 L:D rats increased, their alcohol consumption. Opposingly, male 12:12 L:D rats increased, while male 6:6 L:D rats decreased, their alcohol consumption [29]. This is contrary to the evidence highlighted above that mice and rats of both sexes experience the same effects of shifting diurnal cycles on alcohol consumption. Conflicting reports are not surprising, however, as sex differences in alcohol consumption are sensitive to the rodent strain used [30,31]. Herein, the purpose of this study was to investigate for the first time whether circadian rhythm disruptions that are produced through genetic manipulations (i.e., Clock^Δ19/Δ19^ mutation) interacting with the environment (i.e., shifting diurnal cycles) produce sex-dependent changes in alcohol drinking behaviour in C57Bl/6 mice. Based on past literature, we expect that modifying the *Clock* gene to disrupt circadian rhythms will increase alcohol consumption and preference, and that females will drink more than males. We further hypothesize that a moderate shortening of diurnal cycles (12:12 to 10:10) will increase alcohol consumption and that additional sex differences in this effect will not be observed.

## 2. Materials and Methods

### 2.1. Animals

Litter- and age-matched female and male WT (N = 10 per sex) and homozygous Clock^Δ19/Δ19^ mutant (Females: N = 10; Males: N = 8) C57Bl/6J mice were obtained from a breeding colony maintained at the University of Guelph [32,33]. WT and Clock^Δ19/Δ19^ mice were pair-housed in cages with perforated dividers to avoid the confounding effects of social isolation [34] while ensuring mice accessed only their own food, water, and alcohol. Animals were maintained in a constant environment with an ambient temperature of 21 ± 2 °C, circulating air, and constant humidity of 50 ± 10%. Once the mice were seven weeks old, daily handling and data collection started. Following the 24-day study period, mice were euthanized using CO_2_. All experiments were approved by the University of Guelph Institutional Animal Care and Use Committee and performed in accordance with the guidelines set forth by the Canadian Council on Animal Care (1993).

### 2.2. Diurnal Cycles

**Figure 1:**
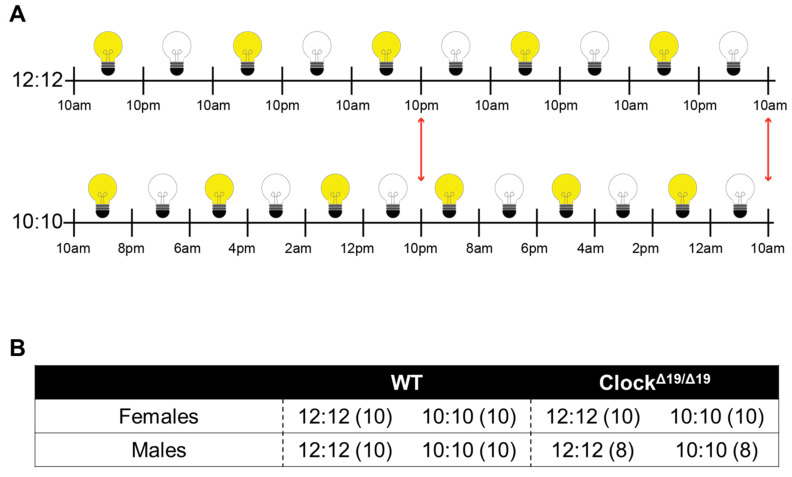
(**A**) Schematic illustrating induced diurnal disruptions through differential light:dark (L:D) cycles in the two study groups. Clear light bulbs signify periods of darkness and yellow light bulbs represent periods of light exposure. After the initial 2.5 days, the two groups are in complete desynchrony (light phase in the 10:10 L:D group, and dark phase in the 12:12 L:D phase; illustrated by the leftmost red double-ended arrow). After the subsequent 2.5 days, the two groups are back in complete synchrony (both begin their light phase; illustrated by the rightmost red double-ended arrow). (**B**) Overview of Study Design; WT (N = 20M, N = 20F) and Clock^Δ19/Δ19^ mice (N = 16M, N = 20F) were pair-housed with a divider in between. Alcohol drinking, food consumption, and body weight were assessed in male and female WT and Clock^Δ19/Δ19^ mice under either a normal 12 h:12 h L:D cycle or a 10 h:10 h L:D cycle.

Mouse cages were housed in custom-built cabinets with regulated house lights to maintain mice on either a standard (12:12) L:D or a disrupted (10:10) L:D cycle (N = 8 or 10 per group, Figure 1A,B). All mice were habituated to a 12:12 L:D cycle for one week prior to the start of the study period. House lights for the 12:12 L:D mice turned on at 10 a.m. (Zeitgeber time 0, ZT0) and off at 10 p.m. (ZT12) each day. The on/off times for the 10:10 L:D group were manually programmed each day using a timer switch controlling the house lights (Figure 1A).

### 2.3. Two-Bottle Choice Paradigm

Alcohol drinking was assessed using the two-bottle choice paradigm as previously described [35,36]. Each mouse accessed two 50 mL graduated plastic bottles topped with stainless-steel drinking spouts. One bottle contained 10% alcohol (*v/v*) and the other contained tap water. The position of each bottle was rotated daily to avoid the confounding effects of spatial preference. Alcohol and water, as well as food, was provided ad libitum throughout the 24-day study period.

### 2.4. Daily Measures

The mass of the alcohol, water, food, and mice was measured daily (in grams [g]). The first two to four days of the 24-day study period constituted baseline measurements of water consumption, without alcohol present. Alcohol was available to mice after the second day of baseline measurements. Daily changes were calculated as the difference between the mass of the alcohol, water, food, and mice present at the time of measurement and the corresponding measurement from the day before. Individual consumption rates were computed as proportional to the kilogram (kg) mass of the mouse (e.g., g/kg of body mass) to correct for the baseline differences in body weights between the groups. The amount of alcohol consumed was calculated as 10% of the daily change in mass for the bottle containing ethanol. Caloric intake was calculated knowing standard mouse chow contains 13,816 J/kg (3.3 Cal/g) of digestible energy with 20% protein, 3% fat, and 77% carbohydrates, and a bottle of 10% ethanol has a caloric value of 2931 J/kg (0.7 Cal/g). Alcohol preference (*P*) was represented as a percentage and calculated using the following formula:

$$P=\frac{\text{alcohol consumed}[\frac{g}{kg}]-\text{water consumed}[\frac{g}{kg}]}{\text{total fluids consumed}[\frac{g}{kg}]}\times 100\%$$

### 2.5. Statistical Analysis 

Statistical analysis was performed using Jeffrey’s Amazing Statistics Program v0.14.1.0 (JASP, University of Amsterdam, Amsterdam, The Netherlands). Within- (days) and between- (sex, genotype, L:D cycle) group comparisons (Figure 1B) were assessed using a two-way repeated measures ANOVA with Tukey’s Honestly Significant Difference post hoc test. Correlational analyses were performed using a one-way Pearson’s r correlation coefficient and shuffled water consumption data were produced by ordered list randomization (using random.org/lists). A significance level of *p* ≤ 0.05 was used. Greenhouse–Geisser corrections were used when Mauchly’s test of sphericity indicated the assumption of sphericity was violated. Data are expressed as mean ± standard error of the mean (SEM). Erroneous raw data points were removed and replaced with an average of the adjacent datapoints. Figures depict the 20-day period during which alcohol was available to mice out of the 24-day study period.

## 3. Results

### 3.1. Alcohol Consumption

**Figure 2:**
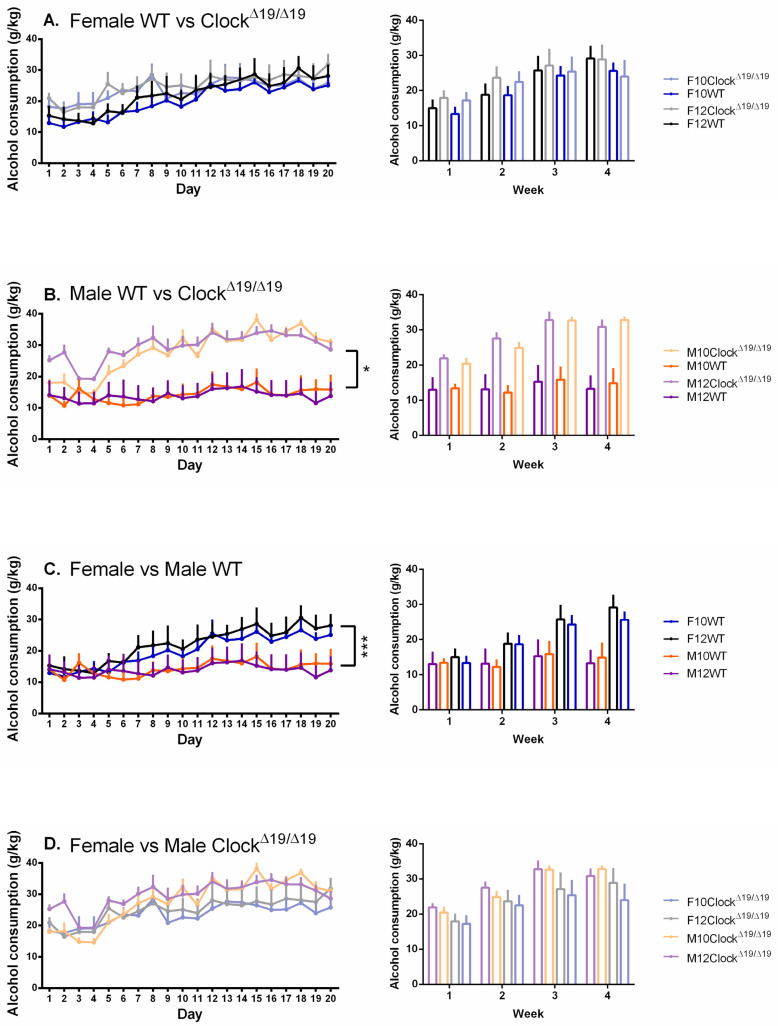
The amount of alcohol consumed by mice is influenced by sex, genotype, and number of drinking days, but not L:D cycles. (**A**). Female WT and Clock^Δ19/Δ19^ mice, regardless of L:D cycles, showed no differences in the amount of alcohol consumed over time. (**B**). Over time, the amount of alcohol consumed by male Clock^Δ19/Δ19^ mice increased such that they were consuming more than the male WT mice for most of the study period. (**C**). The amount of alcohol consumed by female mice diverged from that of male WT mice across the 20-day testing period. (**D**). Female and male Clock^Δ19/Δ19^ mice demonstrated similar amounts of alcohol consumed across the 20-day testing period. F = females, M = males; 12 = 12:12 L:D cycle, 10 = 10:10 L:D cycle; WT = wild-type. Darker tones are WT mice and lighter tones are Clock^Δ19/Δ19^ mice. Black = female 12:12 L:D group, blue = female 10:10 L:D group, purple = male 12:12 L:D group, orange = male 10:10 L:D group. * = *p* < 0.05, *** = *p* < 0.001.

A significant main effect of genotype on alcohol consumption was observed (F (1,68) = 16.139, *p* = 0.000, η_p_^2^ = 0.192) and post hoc comparisons revealed that Clock^Δ19/Δ19^ mice consumed more alcohol than WT mice. A significant sex by genotype interaction was observed (F (1, 68) = 6.481, *p* = 0.013, η_p_^2^ = 0.087) and post hoc comparisons revealed that, while a statistically significant difference between female WT and Clock^Δ19/Δ19^ mice was not observed (Figure 2A), male Clock^Δ19/Δ19^ mice drank significantly more than male WT mice (Figure 2B). A significant sex by genotype by day interaction was also observed (F (4.083, 277.651) = 5.313, *p* = 0.000, η_p_^2^ = 0.072) with post hoc comparisons revealing that alcohol consumption by female WT mice diverged from male WT mice toward the end of the study (Figure 2C) whereas female and male Clock^Δ19/Δ19^ mice consumed similar amounts of alcohol throughout the study (Figure 2D).

### 3.2. Alcohol Preference

**Figure 3:**
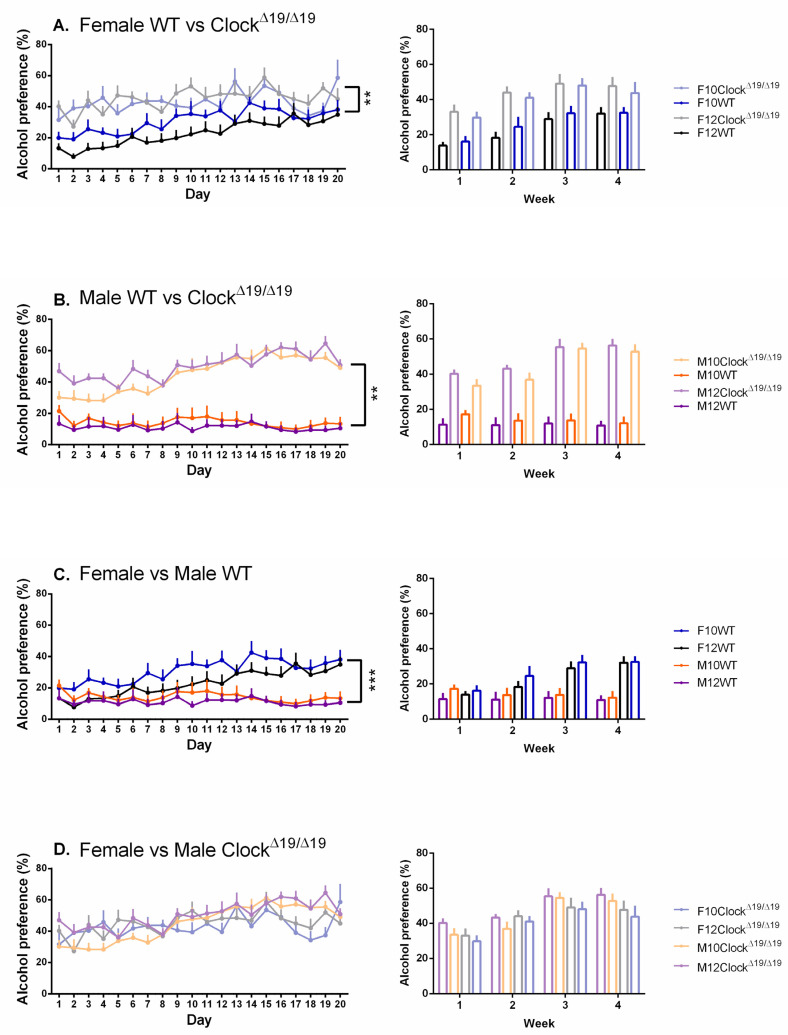
Alcohol preference in mice is influenced by sex, genotype, and number of drinking days, but not L:D cycle. (**A**). Compared to female WT mice, female Clock^Δ19/Δ19^ mice demonstrated enhanced alcohol preference. Over time, female WT alcohol preference increased such that by day 20 they were no longer different from their Clock^Δ19/Δ19^ counterparts. (**B**). Male Clock^Δ19/Δ19^ mice demonstrated enhanced alcohol preference compared to male WT mice, a difference that was maintained across the 20-day testing period. (**C**). Compared to male WT mice, female WT mice demonstrated alcohol preferences that increased over time such that by day 20 the difference between male and female mice was statistically significant. (**D**). Clock^Δ19/Δ19^ mice exhibited alcohol preferences that were not influenced by sex or L:D cycle. F = females, M = males; 12 = 12:12 L:D cycle, 10 = 10:10 L:D cycle; WT = wild-type. Darker tones are WT mice and lighter tones are Clock^Δ19/Δ19^ mice. Black = female 12:12 L:D group, blue = female 10:10 L:D group, purple = male 12:12 L:D group, orange = male 10:10 L:D group. ** = *p* < 0.01, *** = *p* < 0.001.

A significant main effect of genotype on alcohol preference was observed (F (1, 67) = 126.056, *p* < 0.001, η_p_^2^ = 0.653) and post hoc comparisons revealed that Clock^Δ19/Δ19^ mice exhibited greater alcohol preferences compared to WT mice (Figure 3A,B). A significant sex by genotype interaction was also observed (F (1, 67) = 8.818, *p* = 0.004, η_p_^2^ = 0.116) and post hoc comparisons revealed that, whereas both sexes of Clock^Δ19/Δ19^ mice had greater alcohol preferences than their WT counterparts, female WT mice exhibited significantly greater alcohol preferences compared to male WT mice (Figure 3C) while female and male Clock^Δ19/Δ19^ mice exhibited similar alcohol preferences (Figure 3D). A main effect of day was also observed (F (7.835, 524.959) = 12.706, *p* < 0.001, η_p_^2^ = 0.159). A significant sex by genotype by day interaction was found (F (7.835, 524.959) = 5.496, *p* < 0.001, η_p_^2^ = 0.076) and post hoc comparisons revealed that, over the study period, the differences decreased between female WT and Clock^Δ19/Δ19^ mice (Figure 3A), increased between male WT and Clock^Δ19/Δ19^ mice (Figure 3B), and increased between female and male WT mice (Figure 3C). Differences were not observed between female and male Clock^Δ19/Δ19^ mice (Figure 3D). 

### 3.3. Water Consumption

**Figure 4:**
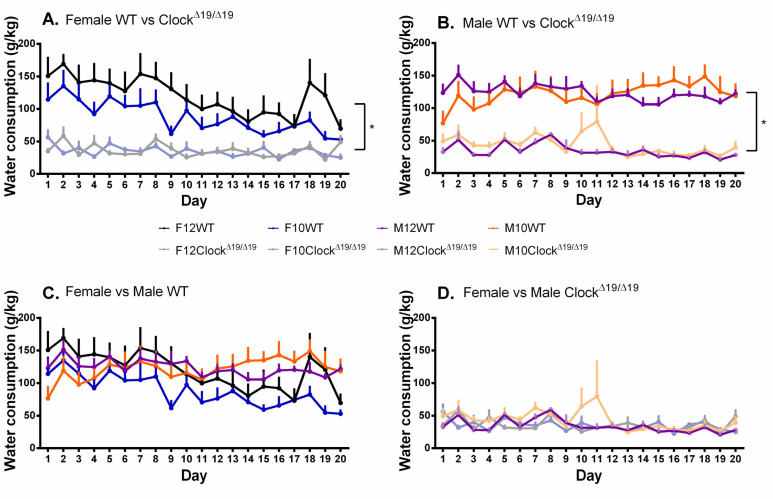
The amount of water consumed by mice is influenced by sex, genotype, and number of drinking days, but not L:D cycle. (**A**). Female Clock^Δ19/Δ19^ mice demonstrated reduced water consumption compared to female WT mice, which demonstrated a decrease in the amount of water consumed across the 20-day testing period such that by the end of the study they were no longer different from female Clock^Δ19/Δ19^ mice. (**B**). Compared to male WT mice, male Clock^Δ19/Δ19^ mice demonstrated reduced amounts of water consumed, an effect that was maintained throughout the duration of the study. (**C**). Female and male WT mice initially demonstrated similar amounts of water consumed, whereas toward the end of the study the female WT mice were consuming slightly less than male WT mice. (**D**). Female and male Clock^Δ19/Δ19^ mice demonstrated similar amounts of water consumed across the 20-day testing period. F = females, M = males; 12 = 12:12 L:D cycle, 10 = 10:10 L:D cycle; WT = wild-type. Darker tones are WT mice and lighter tones are Clock^Δ19/Δ19^ mice. Black = female 12:12 L:D group, blue = female 10:10 L:D group, purple = male 12:12 L:D group, orange = male 10:10 L:D group. * = *p* < 0.05.

A significant main effect of genotype on water consumption was observed (F (1, 68) = 121.934, *p* < 0.001, η_p_^2^ = 0.642) and post hoc comparisons revealed that WT mice consumed much more water than Clock^Δ19/Δ19^ mice (Figure 4A,B). A significant main effect of day on water consumption was also observed (F (7.203, 489.824) = 4.524, *p* < 0.001, η_p_^2^ = 0.062). A significant sex by genotype by day interaction was found (F (7.203, 489.824) = 2.605, *p* = 0.011, η_p_^2^ = 0.037) and post hoc comparisons revealed that female Clock^Δ19/Δ19^ mice began consuming significantly less water than female WT mice on the first day of alcohol consumption, a difference that disappeared toward the end of the study as female WT mice drank less water throughout; male Clock^Δ19/Δ19^ mice also drank significantly less water compared to male WT mice toward the end of the study (Figure 4A,B). Within each genotype group, female and male mice consumed similar amounts of water throughout the study (Figure 4C,D).

### 3.4. Total Fluid Consumption

**Figure 5:**
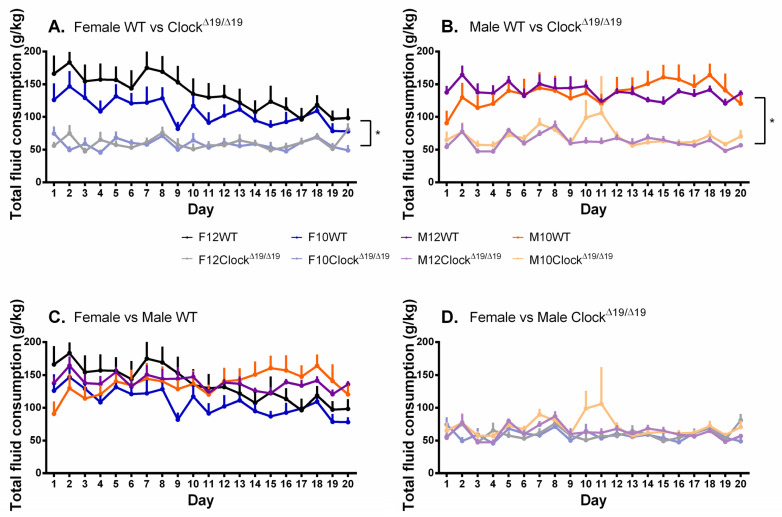
The total amount of fluids consumed by mice is influenced by sex, genotype, and number of drinking days, but not L:D cycle. (**A**). Female Clock^Δ19/Δ19^ mice consumed less fluids in total compared to female WT mice, which demonstrated a decrease in the quantity of total fluids consumed across the 20-day testing period such that by the end of the study they were no longer different from female Clock^Δ19/Δ19^ mice. (**B**). Compared to male WT mice, male Clock^Δ19/Δ19^ mice demonstrated reduced amounts of total fluids consumed, an effect that was maintained throughout the duration of the study. (**C**). Female and male WT mice initially demonstrated similar amounts of total fluids consumed; toward the end of the study, female WT mice were consuming slightly less than male WT mice. (**D**). Female and male Clock^Δ19/Δ19^ mice demonstrated similar amounts of total fluids consumed across the 20-day testing period. F = females, M = males; 12 = 12:12 L:D cycle, 10 = 10:10 L:D cycle; WT = wild-type. Darker tones are WT mice and lighter tones are Clock^Δ19/Δ19^ mice. Black = female 12:12 L:D group, blue = female 10:10 L:D group, purple = male 12:12 L:D group, orange = male 10:10 L:D group. * = *p* < 0.05.

A significant main effect of genotype was observed (F (1, 68) = 96.094, *p* < 0.001, η_p_^2^ = 0.586) and post hoc comparisons revealed that WT mice consumed more fluids overall than Clock^Δ19/Δ19^ mice (Figure 5A,B). A significant main effect of day was also observed (F (7.651, 520.284) = 4.421, *p* < 0.001, η_p_^2^ = 0.057). A significant sex by day interaction was found (F (7.651, 520.284) = 2.221, *p* = 0.027, η_p_^2^ = 0.032) and post hoc comparisons revealed that female mice were drinking significantly less toward the end of the study than they were at the start of the study; an effect that was largely only observed for female mice (Figure 5A). A significant sex by genotype by day interaction was also observed (F (7.651, 520.284) = 2.654, *p* = 0.008, η_p_^2^ = 0.038) and post hoc comparisons revealed that female WT mice drank significantly greater amounts compared to female Clock^Δ19/Δ19^ mice in the first 10 days of the study, with these differences disappearing in the second 10 days, while male WT mice drank significantly greater amounts consistently throughout the study (Figure 5A,B). Within each genotype group, female and male mice consumed similar amounts of fluid throughout the study (Figure 5C,D). 

### 3.5. Food Consumption

**Figure 6:**
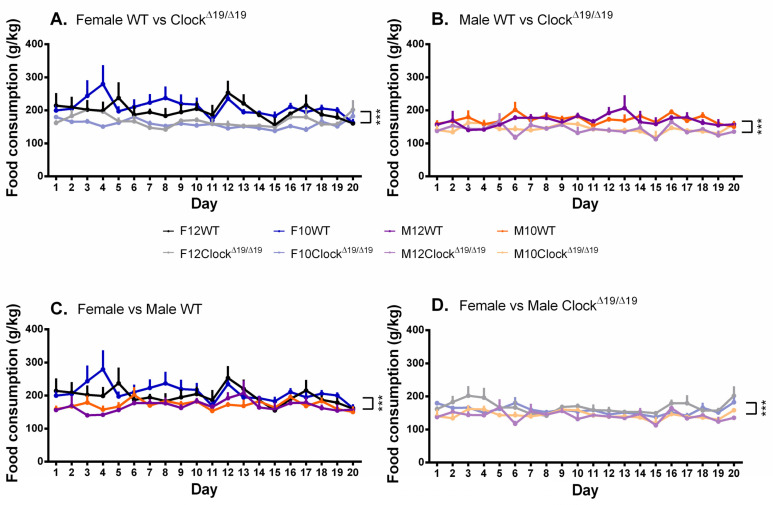
The amount of food consumed by mice is influenced by sex and genotype. (**A**). Female WT mice consumed more food across the 20-day testing period than female Clock^Δ19/Δ19^ mice. (**B**). Male WT mice consumed more food across the 20-day testing period than male Clock^Δ19/Δ19^ mice. (**C**). Female WT mice consumed more food than male WT mice across the 20-day testing period. (**D**). Female Clock^Δ19/Δ19^ mice consumed more food than male Clock^Δ19/Δ19^ mice across the 20-day testing period. F = females, M = males; 12 = 12:12 L:D cycle, 10 = 10:10 L:D cycle; WT = wild-type. Darker tones are WT mice and lighter tones are Clock^Δ19/Δ19^ mice. Black = female 12:12 L:D group, blue = female 10:10 L:D group, purple = male 12:12 L:D group, orange = male 10:10 L:D group. *** = *p* < 0.001.

A significant main effect of genotype on food consumption was observed (F (1,68) = 45.435, *p* < 0.001, η_p_^2^ = 0.401) and post hoc comparisons revealed that WT mice consumed more food than Clock^Δ19/Δ19^ mice (Figure 6A,B). A significant main effect of sex on food consumption was found (F (1,68) = 27.334, *p* < 0.001, η_p_^2^ = 0.287) and post hoc comparisons revealed that female mice consumed more food than male mice throughout the study (Figure 6C,D). Significant interaction effects were not observed.

### 3.6. Caloric Intake

**Figure 7:**
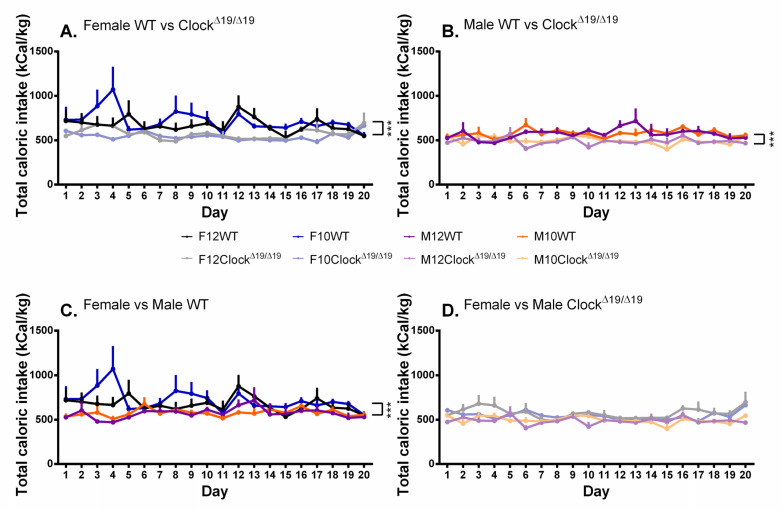
The total caloric intake by mice is influenced by sex and genotype but not L:D cycle. (**A**). Female WT mice consumed fewer calories than female Clock^Δ19/Δ19^ across the 20-day testing period. (**B**). Male WT mice consumed fewer calories than male Clock^Δ19/Δ19^ mice across the 20-day testing period. (**C**). Female WT mice consumed more calories than male WT mice across the 20-day testing period. (**D**). Female and male Clock^Δ19/Δ19^ mice demonstrated similar amounts of calories consumed. F = females, M = males; 12 = 12:12 L:D cycle, 10 = 10:10 L:D cycle; WT = wild-type. Darker tones are WT mice and lighter tones are Clock^Δ19/Δ19^ mice. Black = female 12:12 L:D group, blue = female 10:10 L:D group, purple = male 12:12 L:D group, orange = male 10:10 L:D group. *** = *p* < 0.001.

A significant main effect of genotype on caloric intake was observed (F (1,39) = 29.510, *p* < 0.001, η_p_^2^ = 0.431) and post hoc comparisons revealed that, overall, WT mice consumed fewer calories than Clock^Δ19/Δ19^ mice (Figure 7A,B). A significant main effect of sex was observed (F (1,39) = 21.171, *p* < 0.001, η_p_^2^ = 0.352) and post hoc comparisons revealed that overall male mice consumed fewer calories than female mice (Figure 7A,B). Differences in caloric intake were not observed between females and males within each genotype group (Figure 7C,D).

### 3.7. Body Weight

**Figure 8:**
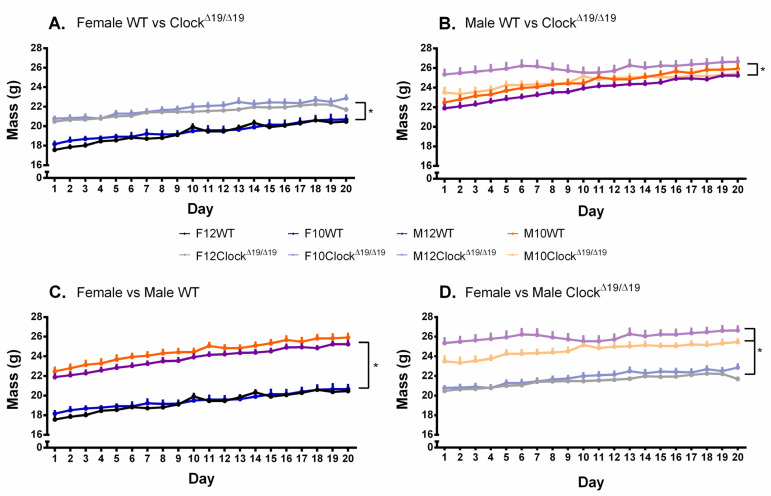
Changes in mouse body weight over time are influenced by sex, genotype, number of drinking days, and L:D cycle. (**A**). Female WT mice demonstrated greater body weights compared to female Clock^Δ19/Δ19^ mice; an effect most prominent in female 12:12 WT mice. (**B**). Male WT mice demonstrated greater body weights compared to male Clock^Δ19/Δ19^ mice; an effect most prominent in male 12:12 WT mice. (**C**). Female and male WT mice demonstrated similar body weights, with the greatest observable differences being in the female 12:12 mice and male 10:10 mice. (**D**). Female and male Clock^Δ19/Δ19^ mice demonstrated similar body weights, with the greatest observable differences being in the male 12:12 and 10:10 mice. F = females, M = males; 12 = 12:12 L:D cycle, 10 = 10:10 L:D cycle; WT = wild-type. Darker tones are WT mice and lighter tones are Clock^Δ19/Δ19^ mice. Black = female 12:12 L:D group, blue = female 10:10 L:D group, purple = male 12:12 L:D group, orange = male 10:10 L:D group. * = *p* < 0.05.

A significant main effect of genotype on body weight was found (F (1,68) = 39.940, *p* < 0.001, η_p_^2^ = 0.370) with post hoc comparisons revealing that WT mice gained more weight than Clock^Δ19/Δ19^ mice throughout the study (Figure 8A,B). A significant main effect of sex on body weight was found (F (1, 68) = 245.930, *p* < 0.001, η_p_^2^ = 0.783) with post hoc comparisons revealing that male mice gained more weight than female mice (Figure 8C,D). A significant sex by genotype interaction was observed (F (1, 68) = 4.031, *p* = 0.049, η_p_^2^ = 0.056) and post hoc comparisons showed that female and male Clock^Δ19/Δ19^ mice gained more weight than female and male WT mice, while male WT mice gained more weight than female WT mice (Figure 8A–C). A significant main effect of day (F (2.419, 164.504) = 121.377, *p* < 0.001, η_p_^2^ = 0.641) was also observed. A significant genotype by L:D cycle by day interaction was also observed (F (2.419, 164.504) = 3.399, *p* = 0.028, η_p_^2^ = 0.048); post hoc comparisons revealed that differences were most apparent between WT 12:12 L:D mice and Clock^Δ19/Δ19^ 10:10 L:D mice after day 10 of testing, but only for comparisons with subsequent testing days, likely reflective of the increase in body weight reported in WT mice (Figure 8A,B). It is interesting to note that body weight seems to be the only measure in this study that demonstrated a compounding effect of genotype and diurnal cycle circadian rhythm disruption.

## 4. Discussion

This study aimed to investigate the effect of possible gene and environment interactions on alcohol consumption and preference in female and male mice, and found that genetic, but not environmental, circadian rhythm disruptions sex-dependently influence alcohol consumption and preference in mice (results summarized in Table 1). Female WT mice developed increased alcohol consumption compared to male WT mice approximately one week after the initiation of alcohol drinking. Clock^Δ19/Δ19^ mice, on the other hand, did not display these sex differences and consistently consumed more alcohol than WT mice. Alcohol consumption and preference in female WT mice increased until they were no longer different from female Clock^Δ19/Δ19^ mice. Male WT mice, however, consistently consumed smaller amounts of alcohol and exhibited reduced alcohol preference compared to male Clock^Δ19/Δ19^ mice. Shifting diurnal cycles did not alter alcohol consumption or preference in our study.

Our findings are consistent with previous studies demonstrating that female rodents consistently consume more alcohol than male rodents [31,37,38,39,40,41]. Sex-dependent differences in alcohol consumption and preference are expected given reported sex differences in ethanol metabolism and pharmacokinetics [42], taste reactivity [43], as well as sexually dimorphic mechanisms in the dopaminergic reward pathways through which ethanol has an effect [44,45]. Moreover, sex-dependent differences between Clock^Δ19/Δ19^ and WT mice are likely, given that the SCN innervates and regulates the circadian release of glucocorticoids from brain regions associated with neuroendocrine function; moreover, glucocorticoids reciprocally regulate the circadian rhythm [28,46,47]. However, changes in alcohol consumption can also occur independent of gonadal hormones [31]. 

The lack of any observable sex differences in the Clock^Δ19/Δ19^ mice used in our study contradicts results by Ozburn et al. which demonstrated that, while Clock^Δ19/Δ19^ mice consumed more alcohol, the female Clock^Δ19/Δ19^ mice exhibited a greater magnitude of increase compared to males [27]. These contradictory findings may be attributed to differences between the mouse strains used as the background strain by the two studies: we used mice maintained on a C57BL/6J background, whereas Ozburn et al. used mice maintained on a BALB/c background. Comparing voluntary alcohol drinking via the two-bottle choice paradigm across 10–20 inbred mouse strains repeatedly demonstrates that C57BL/6J mice consume the greatest amount of alcohol compared to all other strains investigated, which might have also impacted our ability to detect the additional impact of genetics and sex on this phenotype [48,49]. The high levels of drinking in our female mice may have contributed to a ceiling effect that could obscure any additional impact of the Clock^Δ19/Δ19^ mutation on alcohol drinking; testing a range of alcohol concentrations would help to mitigate this potential confound, like the range of escalating ethanol concentrations used by Ozburn et al. (from 3% up to a maximum of 21% ethanol [v/v]) [31]. We did, however, observe that the difference in alcohol preference between female WT and Clock^Δ19/Δ19^ mice lessened throughout the study, whereas the difference between male WT and Clock^Δ19/Δ19^ mice remained consistent. These observations warrant future investigations of sex differences in Clock^Δ19/Δ19^ mice, possibly using a wider dose range of ethanol. The Clock^Δ19/Δ19^ mice in our study also had markedly higher alcohol preference at the start of the study, and this magnitude of effect was maintained throughout the study period. This likely reflects the increased reward sensitivity and drug-seeking behaviours commonly observed in Clock^Δ19/Δ19^ mice [50,51] and further supports the Clock protein as an influential factor in driving problematic alcohol consumption, as is surmised from the clinical literature [8,52]. Indeed, mutating the *Clock* gene in mice increases dopamine release and turnover in the striatum, increasing dopaminergic tone and altering dopamine receptor expression [53], likely leading to the enhanced reward sensitivity commonly reported. With widespread changes in dopamine-receptor expression occurring during adolescence, and evidence that modelling adolescent “binge drinking” in mice sex-dependently increases alcohol consumption in adulthood, it may be worthwhile to investigate changes in *Clock* expression during adolescence in relation to alcohol drinking [39,54,55]; especially since shifting diurnal cycles in male adolescent mice also increases alcohol consumption [56].

Our finding that housing mice on a shortened (10:10) L:D cycle did not affect alcohol consumption and preference is surprising, and somewhat inconsistent with existing evidence of increased or decreased alcohol consumption resulting from the various models of disrupted diurnal cycling mentioned above [13,14,29,57]. While our decision to use the 10:10 L:D model of shifting diurnal cycles was based on past evidence of resulting circadian desynchrony and concomitant changes in physiological processes [58], this disruption may be insufficient to produce robust changes in alcohol drinking. Future studies may benefit from using a model with a greater degree of disruption (e.g., 6:6 instead of 10:10 L:D) to produce a more robust effect on alcohol drinking [59]. However, this moderate disruption is more akin to disruptions that might happen due to travel or daylight savings and may suggest that moderate circadian disruptions do not impact alcohol drinking, especially compared to more drastic disruptions. Thus, the results of our genetic and environmental investigation reveal that it may be the genetic, and not environmental, disruption that is largely influencing alcohol drinking behaviour. Since our environmental manipulation did not alter alcohol drinking significantly by itself, it is possible that our design prevents us from detecting any gene–environment interactions. Furthermore, the extent of effect of the gene mutation on alcohol drinking may also preclude the detection of such an interaction due to a possible ceiling effect on alcohol drinking. Therefore, it might be important for future studies to investigate whether such an interaction can be observed at a molecular level by characterizing Clock protein expression levels over time as a possible confounding factor, especially given evidence from Ozburn et al. that ethanol exposure reduces *Clock* expression [27]. Clinical investigations report reduced baseline Clock mRNA levels in patients with alcohol use disorder, as well reduced baseline mRNA levels of circadian proteins BMAL1, Per1, Per2, Cry1, and Cry2 [60]. Per2 mutation-induced circadian rhythm disruptions are also linked to increased alcohol intake in both mice and humans [61,62,63,64] and Per1, Per2 and Bmal1 expression levels are disrupted in participants undergoing a simulated night shift work schedule [65]. Recently, selective ablation of Bmal1 and Per2 from medium spiny neurons in the mouse striatum enhanced alcohol consumption in males and reduced alcohol consumption in females, while only the effect in males was observed after ablation of Per2 [66].

Comparing measures of alcohol, food, and water consumption, as well as total fluid and caloric intake, reciprocal patterns to those described for alcohol consumption were first noted in the amount of water consumed by female WT mice throughout the study. Female WT mice initially drank much more water than male WT mice but, with a concomitant increase in alcohol consumption, ended the study drinking no more water than the minimal amount consumed by female and male ClockΔ19 mice; a finding that is supported by existing evidence of rats maintaining a constant quantity of fluids consumed daily and an inverse relationship between water and alcohol during free-access choice paradigm testing [35,36]. Male WT mice consistently drank more water than male ClockΔ19 mice throughout the study. Undisrupted water consumption after shifting diurnal cycles in males is previously reported in C57BL/6J mice that demonstrated increased intermittent alcohol consumption after being exposed to alternating standard and lengthened diurnal cycle [67]. Second, we noted that the amount of food consumed by female WT mice decreased while alcohol consumption increased, while their total caloric intake remained relatively constant throughout the study. This seems to indicate that female mice were altering their food intake to maintain a constant caloric intake as they consumed additional calories from alcohol. This observation is supported by previous evidence of sex-dependent differences in the maintenance of mouse feeding behaviour related to meal size and by proxy, caloric intake [68].

Although our findings contribute to our understanding of how sex and circadian biology influence alcohol consumption and preference, additional investigations are required to relate the sex differences reported herein with estrous cycle fluctuations of estrogen and progesterone. For example, estrogen, when endogenously applied, shortens the period of circadian rhythmicity evident in the locomotor behaviour of mice [69]. Investigating the role of the estrous cycle may reveal additional patterns in consumptive behaviour. Relatedly, future studies should also examine the minutiae of alcohol consumption (i.e., number of bouts, bout frequency and volume) to relate features of consumptive behaviour with measures of alcohol consumption and preference [70]. We intend to use automated lickometers in a subsequent investigation of Clock^Δ19/Δ19^ mouse drinking behaviour [71]. Further assessing the temporality of alcohol consumption and preference in female and male mice may provide additional knowledge, including whether epochs of increased vulnerability toward alcohol consumption exist; this is especially crucial when considered together with estrous cycle fluctuations of estrogen and progesterone. Finally, the effect of age on sex differences in alcohol consumption and preference changes produced by circadian rhythm disruptions warrants further investigation, because adolescence is period of increased sensitivity to the initiation of alcohol drinking [55], and adolescents demonstrate shifted sleep/wake cycles compared to adult populations [24,72].

## 5. Conclusions

In conclusion, the findings from this study demonstrate that circadian rhythm disruptions influence alcohol consumption and preference in a sex-dependent manner. Future studies will investigate the mechanisms underlying these sex-dependent effects and whether targeting these mechanisms will reduce the impact of circadian rhythm disruptions on alcohol consumption patterns. This is particularly important as much of the workforce engages in shiftwork and reportedly uses problematic alcohol consumption as a mechanism for improving sleep and quality of life.

## Figures and Tables

**Table 1:** A summary table highlighting the reported results across all groups and measures investigated. All female and male values are compared to female or male WT mice on a 12:12 L:D cycle (indicated with “N/A”). F = females, M = males; 12 = 12:12 L:D cycle, 10 = 10:10 L:D cycle; WT = wild-type. Up arrow = increase, down arrow = decrease, horizontal line = no change.

Group	Alcohol Consumption	Alcohol Preference	Water Consumption	Total Fluid Consumption	Food Consumption	Caloric Intake	Weight Gain
F12WT	N/A	N/A	N/A	N/A	N/A	N/A	N/A
F10WT	–	–	–	–	–	–	–
F12Clock^Δ19/Δ19^	↑	↑	↓	↓	↓	↑	↑
F10Clock^Δ19/Δ19^	↑	↑	↓	↓	↓	↑	↑
M12WT	N/A	N/A	N/A	N/A	N/A	N/A	N/A
M10WT	–	–	–	–	–	–	–
M12Clock^Δ19/Δ19^	↑	↑	↓	↓	↓	↑	↑
M10Clock^Δ19/Δ19^	↑	↑	↓	↓	↓	↑	↑

## Data Availability

The data presented in this study are openly available in the Open Science Framework repository at DOI 10.17605/OSF.IO/ZSA5F.
